# Proteomic identification of cryostress in epididymal spermatozoa

**DOI:** 10.1186/s40104-016-0128-2

**Published:** 2016-11-21

**Authors:** Sung Jae Yoon, Md Saidur Rahman, Woo Sung Kwon, Do Yeal Ryu, Yoo Jin Park, Myung Geol Pang

**Affiliations:** Department of Animal Science & Technology, Chung-Ang University, Anseong, Gyeonggi-Do 456-756 Republic of Korea

**Keywords:** Cryopreservation, Cryostress, Protein, Spermatozoa

## Abstract

**Background:**

Cryopreservation of epididymal spermatozoa is important in cases in which it is not possible to collect semen using normal methods, as the sudden death of an animal or a catastrophic injury. However, the freezing and thawing processes cause stress to spermatozoa, including cold shock, osmotic damage, and ice crystal formation, thereby reducing sperm quality. We assessed the motility (%), motion kinematics, capacitation status, and viability of spermatozoa using computer-assisted sperm analysis and Hoechst 33258/chlortetracycline fluorescence staining. Moreover, we identified proteins associated with cryostress using a proteomic approach and performed western blotting to validate two-dimensional electrophoresis (2-DE) results using two commercial antibodies.

**Results:**

Cryopreservation reduced viability (%), motility (%), straight-line velocity (VSL), average path velocity (VAP), amplitude of lateral head displacement (ALH), and capacitated spermatozoa, whereas straightness (STR) and the acrosome reaction increased after cryopreservation (*P* < 0.05). Nine proteins were differentially expressed (two proteins decreased and seven increased) (>3 fold, *P* < 0.05) before and after cryopreservation. The proteins differentially expressed following cryopreservation are putatively related to several signaling pathways, including the ephrinR-actin pathway, the ROS metabolism pathway, actin cytoskeleton assembly, actin cytoskeleton regulation, and the guanylate cyclase pathway.

**Conclusion:**

The results of the current study provide information on epididymal sperm proteome dynamics and possible protein markers of cryo-stress during cryopreservation. This information will further the basic understanding of cryopreservation and aid future studies aiming to identify the mechanism of cryostress responses.

## Background

For decades, sperm cryopreservation has been an important tool for assisted reproductive techniques and storing genetic resources [1, 2]. Using cryopreservation for effective sperm storage has enabled advances in the livestock industry and management of infertility [3, 4]. However, in cases in which it is not possible to collect spermatozoa normally such as unexpected death and catastrophic injury, cryopreservation of epididymal spermatozoa can be advantageous. Therefore, preservation of epididymal spermatozoa can play an important role in human and animal reproduction.

The freezing and thawing processes of sperm cryopreservation inevitably cause structural and functional alterations, thereby reducing fertility [5–7]. Although the bovine is regarded as a model of successful cryopreservation among various species, the process causes up to 50% loss of viable spermatozoa [8].

During the different stages of cryopreservation, spermatozoa are exposed to various types of stress, such as cold shock, osmotic damage, and ice crystal formation [7, 9]. Furthermore, cryopreservation induces the excess production of reactive oxygen species (ROS), disruption of mitochondrial membrane potential, and an apoptosis-like phenomenon in spermatozoa [1, 10]. These changes are associated with alterations in sperm membrane compounds, resulting in dramatic reductions in motility, viability, and fertilizing ability [11].

It is well known that sperm functions and structures are affected by protein degradation and post-translational modifications, such as phosphorylation, during cryopreservation [5, 10]. Research over the past decade has provided compelling evidence that spermatozoa are damaged substantially during cryopreservation, thereby reducing sperm function and semen quality [3, 4]. However, little is known about cryostress at the protein level in spermatozoa. Advances in two-dimensional electrophoresis (2-DE) and mass spectrometry techniques have enabled the identification of proteins related to cryopreservation [12]. Therefore, these methods may facilitate the discovery of biomarkers of cryostress at the proteome level in spermatozoa.

In the present study, bull epididymal sperm cryopreservation was used as a model system of cryopreservation. First, we evaluated various sperm parameters before- and after-cryopreservation. To identify the protein markers related to cryostress, a comprehensive proteomic study of the associated signaling pathways was performed.

## Methods

### Sample collection

Native Korean Bull (Hanwoo) testes were transferred on ice from a local slaughterhouse (Seomun Co., Hwaseong, Korea) to the laboratory within 3 h [13]. Sperm samples were obtained from nine individual bull epididymides. A small cut was made at the cauda epididymis of the testis and backflushed Phosphate-buffered saline (PBS; Sigma-Aldrich, St. Louis, MO, USA) into the end of the vas deferens using a 10-mL syringe. Each collected sample was washed by centrifugation at 700 × *g* for 15 min [14]. To rule out individual difference in resistance to cryo-stress, the nine samples were mixed for the sperm parameter and proteome analyses. All procedures were performed according to the guidelines for the ethical treatment of animals by the Institutional Animal Care and Use Committee of Chung-Ang University, Seoul, Korea.

### Cryopreservation of spermatozoa

Sperm cryopreservation was performed as previously described [15, 16]. Briefly, washed samples were diluted (100 × 10^6^ cells/mL) in Tris-egg yolk buffer (TYB; 250 mmol/L Tris, 88.5 mmol/L citric acid, 68.8 mmol/L glucose, and 20% egg yolk) and cooled to 4 °C over 2 h. Then an equal volume of TYB containing 12% glycerol was added to dilute the sample. After equilibration at 4 °C for 2 h, samples packaged into 0.5-mL straws were frozen in liquid nitrogen vapor for 15 min. Finally, the straws were plunged into liquid nitrogen for long-term storage. Samples were thawed at 37 °C for 1 min after 2 wk of cryopreservation.

### Computer-assisted sperm analysis (CASA)

Sperm motility (%) and kinematics of the samples were analyzed by the method of Kown et al. using CASA system (Sperm Analysis Imaging System version SAIS-PLUS 10.1; Medical Supply, Seoul, Korea) [17]. Briefly, 10 μL of each sample was observed on 37 °C Makler chamber (Makler, Haifa, Israel) using the 10× objective in phase contrast mode. The obtained images were analyzed to assess sperm motility (%), average path velocity (VAP), straight-line velocity (VSL), curvilinear velocity (VCL), wobble (WOB), straightness (STR), linearity (LIN), and amplitude of lateral head displacement (ALH). At least 250 sperm cells were recorded for each sample.

### Assessment of capacitation status by Hoechst 33258 (H33258)/chlortetracycline fluorescence (CTC)

The H33258/CTC dual staining method was performed as previously described [16, 18]. Briefly, 15 μL of H33258 solution (10 μg H33258/mL PBS) was added to 135 μL of the sample. After a 10 min incubation at room temperature (RT), 250 μL of 2% (w/v) polyvinylpyrrolidone (Sigma-Aldrich) in PBS was added to the mixture. Then, the sample was washed by centrifugation at 700 × *g* for 5 min and the pellet was resuspended in 150 μL of PBS and 150 μL of CTC solution (750 mmol/L CTC in 5 μL of buffer composed of 20 mmol/L Tris, 130 mmol/L NaCl, and 5 mmol/L cysteine, pH 7.4).

Capacitation status and viability were analyzed using a Microphot-FXA microscope (Nikon, Tokyo, Japan) under epifluorescent illumination. Four capacitation patterns were observed: live acrosome-reacted (AR), live capacitated (B), live non-capacitated (F), or dead (D). For each sample, at least 400 spermatozoa per slide were evaluated.

### 2DE, gel-image analysis

All procedures followed the methods of Kwon et al. [17]. Samples were centrifuged at 700 × *g* for 15 min with isotonic 45% Percoll in PBS [4, 17, 19]. After rehydrating with 250 μg solubilized protein samples for 12 h at 4 °C, 24-cm-long NL Immobiline DryStrips (pH 3–11; Amersham, Piscataway, NJ, USA) were focused for first-dimension electrophoresis using an IPGphor isoelectric focusing system. After equilibrate the strips, 2-DE was performed using 12.5% (w/v) SDS-polyacrylamide gel electrophoresis (SDS-PAGE) gels. Silver staining was performed and the gels were scanned with a GS-800 calibrated scanner (Bio-Rad, Hercules, CA, USA). To detect, quantify, and match spots and to perform the comparative and statistical analyses, PDQuest 8.0 software (Bio-Rad, Hercules, CA, USA) was used.

### In-gel digestion

Proteins were subjected to in-gel trypsin digestion. Excised gel spots were destained with 100 μL of destaining solution (30 mmol/L potassium ferricyanide, 100 mmol/L sodium thiosulfate) with shaking for 5 min. After removal of the solution, gel spots were incubated with 200 mmol/L ammonium bicarbonate for 20 min. The gel pieces were dehydrated with 100 μL of acetonitrile and dried in a vacuum centrifuge. The procedure was repeated three times. The dried gel pieces were rehydrated with 20 μL of 50 mmol/L ammonium bicarbonate containing 0.2 μg modified trypsin (Promega, Madison, WI, USA) for 45 min on ice. After removing the solution, 70 μL of 50 mmol/L ammonium bicarbonate was added. The digestion was performed overnight at 37 °C. The peptide solution was desalted using a C18 nano column (homemade, Waters Corp., Milford, MA, USA).

### Desalting and concentration

Custom-made chromatographic columns were used for desalting and concentrating the peptide mixture prior to mass spectrometric analysis. A column consisting of 100–300 nL of Poros Reversed-phase R2 material (20–30-μm beads, PerSeptive Biosystems, Framingham, MA, USA) was packed in a constricted GELoader Tip (Eppendorf, Hamburg, Germany). A 10-mL syringe was used to force liquid through the column by applying gentle air pressure. Thirty microliters of the peptide mixture from the digestion supernatant was diluted with 30 μL of 5% formic acid, loaded onto the column, and washed with 30 μL of 5% formic acid. For analyses by tandem mass spectrometry (MS/MS) analyses, peptides were eluted with 1.5 μL of 50% methanol/49% H_2_O/1% formic acid directly into a precoated borosilicate nanoelectrospray needle (EconoTip™, New Objective, Woburn, MA, USA).

### ESI-MS/MS

MS/MS of peptides generated by in-gel digestion was performed by nano-electrospray ionization (ESI) on a MicroQ-TOF III mass spectrometer. The source temperature was RT. A potential of 1 kV was applied to the precoated borosilicate nanoelectrospray needles (EconoTip™, New Objective) in the ion source combined with a nitrogen back-pressure of 0–5 psi to produce a stable flow rate (10–30 nL/min). The cone voltage was 800 V. The quadrupole analyzer was used to select precursor ions for fragmentation in the hexapole collision cell. The collision gas was Ar at a pressure of 6–7 × 10^–5^ mbar and the collision energy was 15–40 V. Product ions were analyzed using an orthogonal time-of-flight (TOF) mass analyzer, fitted with a reflector, a micro-channel plate detector, and a time-to-digital converter. The data were processed using a peptide sequence system.

### Database search

An MS/MS ion search was assigned as the ion search option in MASCOT software (MASCOT version 2.3, Matrix Science, Boston, MA, USA). Peptide fragment files were obtained from the peptide peaks in ESI-MS by ESI-MS/MS. Trypsin was selected as the enzyme, with one potentially missed cleavage site. An ESI-QTOF instrument was used for protein mass determination. The peptide fragment files were used to search the database using the Mascot search engine (Matrix Science), and the results were limited to *Sus scrofa*. Oxidized methionine was set as a variable modification, and carbamidomethylated cysteine was set as a fixed modification. The mass tolerance was set at ±1 and ±0.6 Da for the peptides and fragments, respectively. High scores were defined as those above the default significance threshold in MASCOT (*P* < 0.05, peptide score > 50).

### Signaling pathway

To identify signaling pathways associated with the protein markers, Pathway Studio (v 9.0, Aridane Genomics, Rockville, MD, USA) was used. Differentially expressed proteins were analyzed in Pathway Studio to determine significantly matched pathways for each protein.

### Western blotting

Western blotting was performed as described previously [17], with modifications. Commercial polyclonal anti-SOD2 (Abcam, Cambridge, MA, USA) and polyclonal anti-NUDFV2 (Abcam) were used, and monoclonal anti-α-tubulin (Abcam) was used as a control. Briefly, the samples were washed by centrifugation in DPBS at 10,000 × *g* for 5 min. The pellets were re-suspended with lysis buffer containing 5% 2-mercaptoethanol and incubated for 10 min at RT. The samples were electrophoresed on a 12% SDS-polyacrylamide gel and transferred to polyvinylidene fluoride membranes (Amersham). The membranes were blocked with PBS-Tween containing 5% skim milk powder (blocking solution) for 3 h at RT. After washing, the membranes were incubated overnight with anti-NDUFV2 (1:3,000) and anti-SOD2 (1:5,000) diluted with blocking solution. Then, the membranes were incubated with horseradish peroxidase conjugated anti-rabbit immunoglobulin G (1:3,000, Abcam) for 1 h. After the membranes were washed, proteins were detected by enhanced chemiluminescence reagents. All bands were scanned with a GS-800 Calibrated Imaging Densitometer (Bio-Rad) and analyzed with Quantity One (v.4.6, Bio-Rad). The signal intensity ratios of the bands were calculated for SOD and NUDFV2/α-tubulin.

### Statistical analysis

Data were analyzed with SPSS v.21.0 (SPSS Inc., Chicago, IL, USA). The Student’s two-tailed *t*-test was used to compare the values from before and after cryopreservation after performing normality and variance homogeneity tests. In Pathway Studio, Fisher’s Exact Test was used to determine if the pathways were statistically correlated with differentially expressed proteins. *P* < 0.05 was considered statistically significant. Data are expressed as the mean ± SEM. Fisher’s exact test was used to determine the probability that a protein is involved in a particular signaling pathway (*P* < 0.05).

## Results

### Sperm parameters

**Fig. 1:**
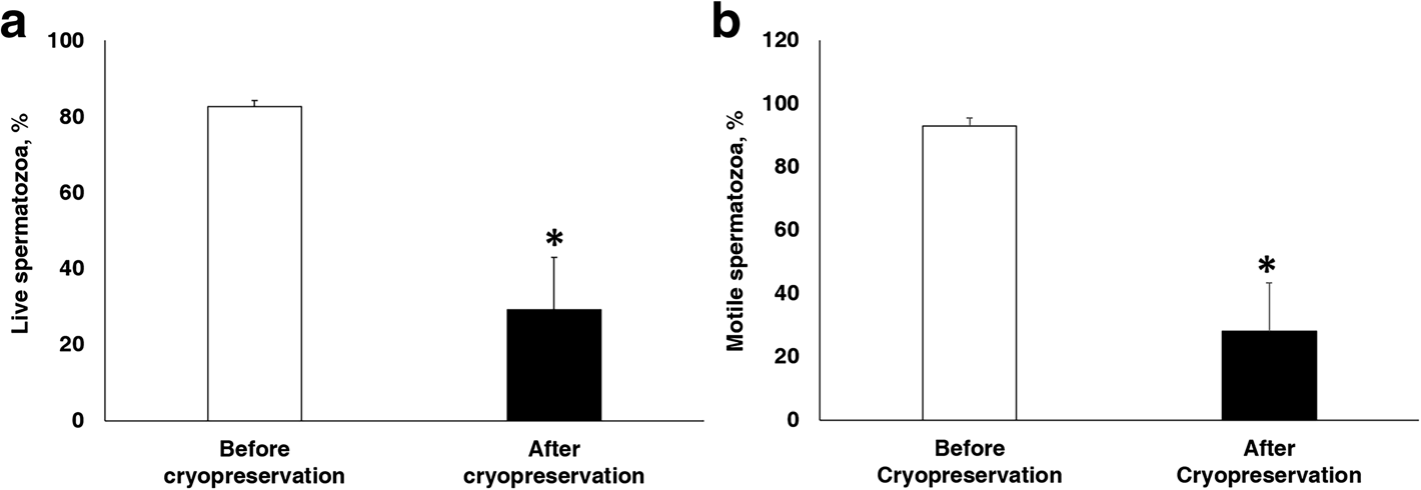
Effect of cryopreservation on sperm. **a** Viability **b** Motility before and after cryopreservation. Data are presented as mean ± SEM. (*P < 0.05, *n* = 9)

**Fig. 2:**
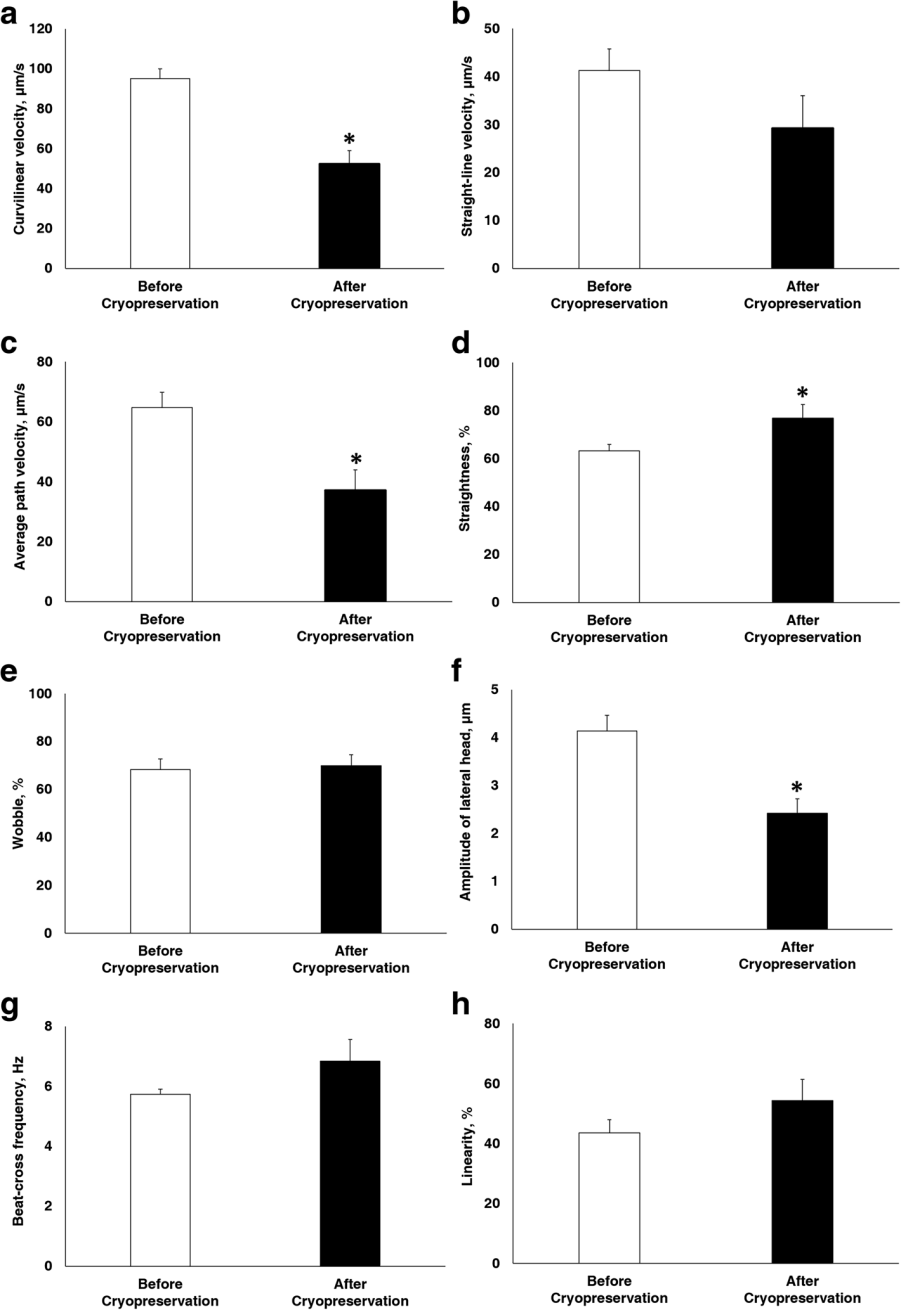
Effect of cryopreservation on sperm motion kinematics. **a** Curvilinear velocity **b** Straight-line velocity **c** Average path velocity **d** Straightness **e** Wobble **f** Amplitude of lateral head **g** Beat-cross frequency **h** Linearity before and after cryopreservation. Data are presented as mean ± SEM. (*P < 0.05, *n* = 9)

**Fig. 3:**
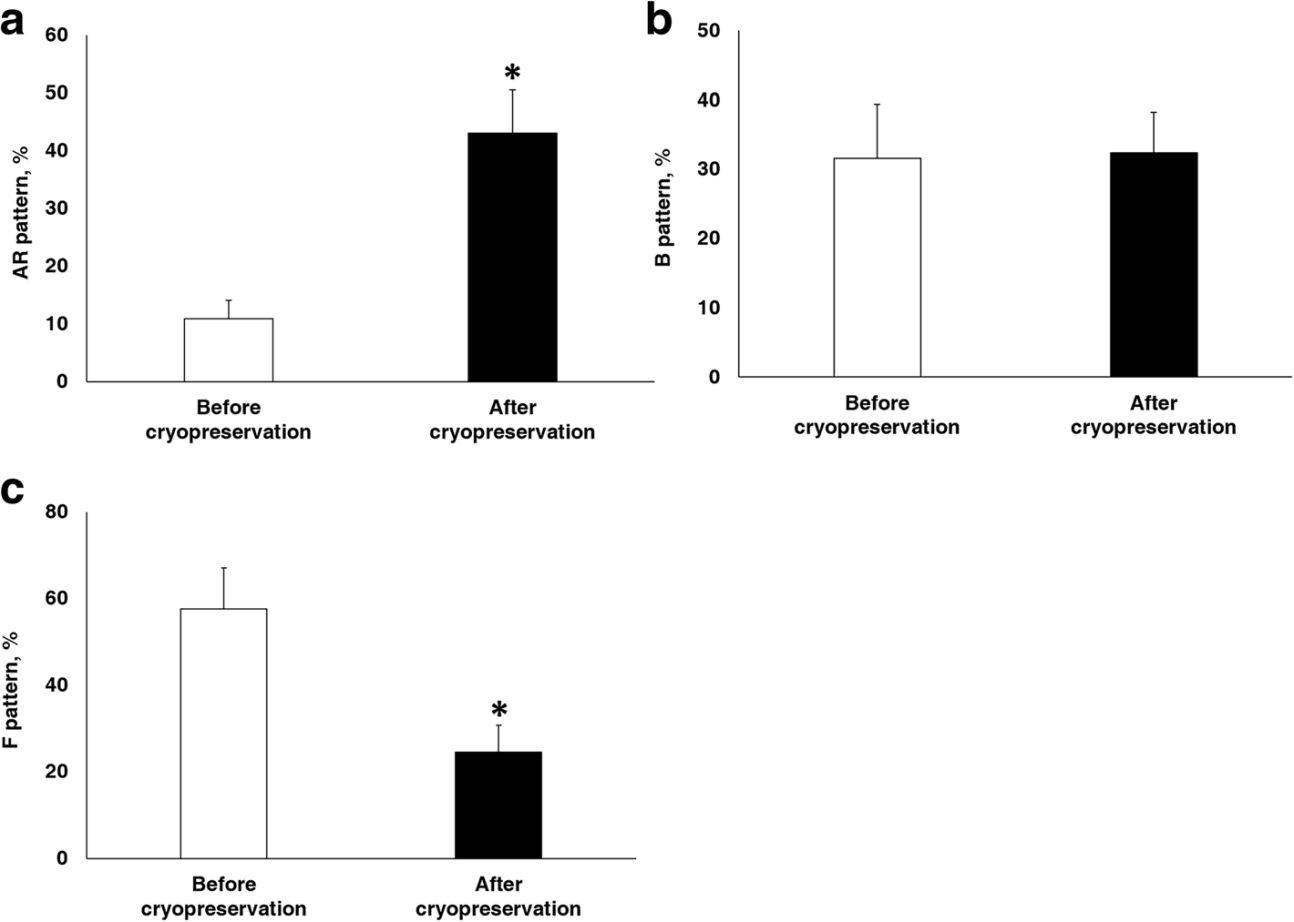
Effect of cryopreservation on live sperm capacitation status. **a** AR pattern. **b** B pattern. **c** F pattern assessed by combined CTC/H33258 staining. Data are presented as mean ± SEM. (*P < 0.05, *n* = 9)

The motility and motion kinematics of spermatozoa before and after cryopreservation were measured by the CASA technique, as described previously [18]. Motility (%), VCL, VAP, and ALH significantly decreased during cryopreservation (Figs. 1 and 2, *P* < 0.05), whereas STR increased significantly (Fig. 2, *P* < 0.05). However, there were no significant differences in VSL, WOB, BCF, or LIN (Fig. 2). To evaluate sperm capacitation status and viability, CTC/H33258 dual staining was performed. The AR pattern increased significantly, whereas the F pattern significantly decreased during cryopreservation (Fig. 3, *P* < 0.05). However, there was no difference in the B pattern (Fig. 3). The viability of frozen-thawed spermatozoa was significantly lower than that of spermatozoa before cryopreservation (Fig. 1, *P* < 0.05).

### 2-DE

**Fig. 4:**
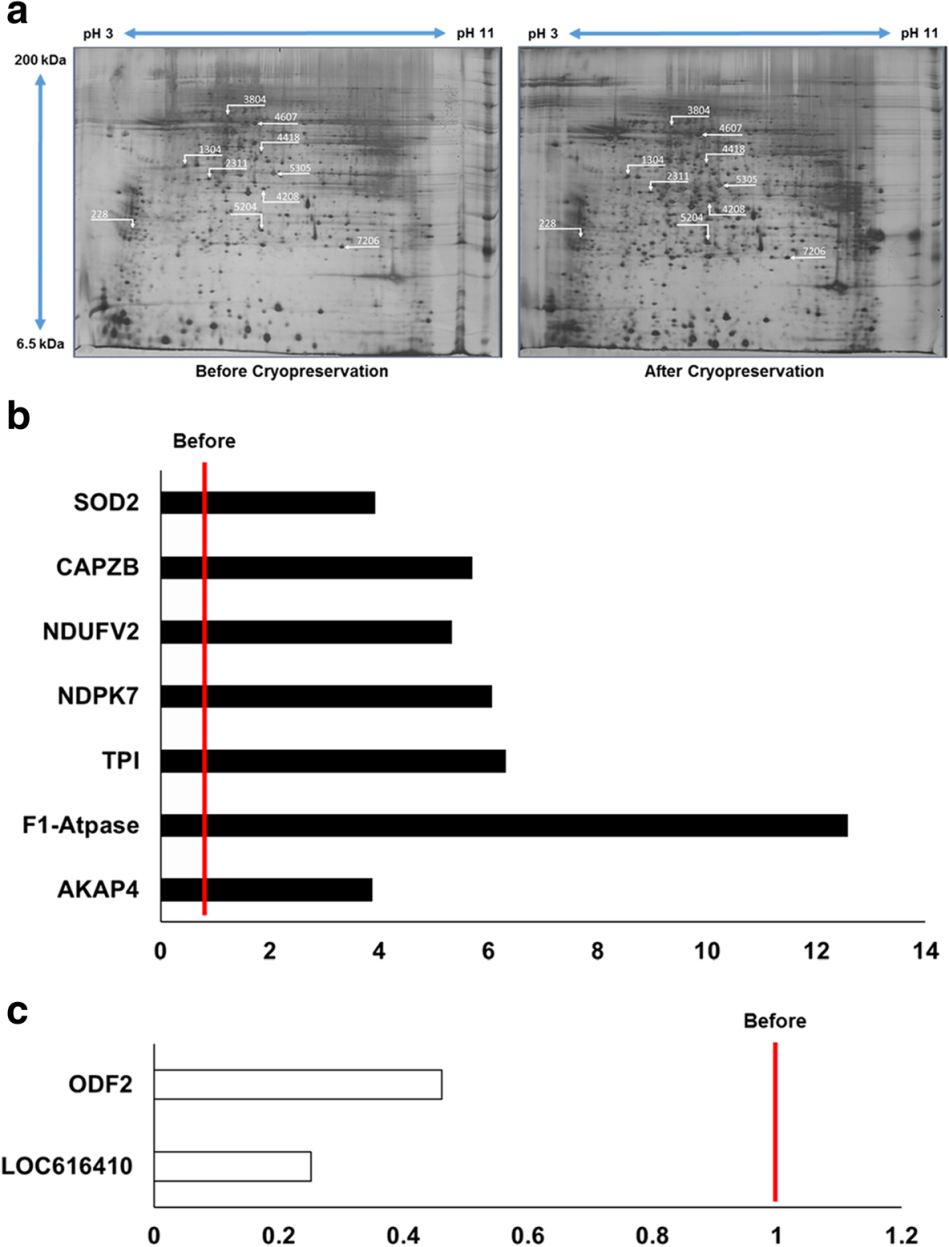
2-DE Separation of proteins by 2-DE. 2-DE gels were stained with silver nitrate and analyzed using PDQuest 8.0 software. Protein spots from (**a**) before cryopreservation and after cryopreservation. **b** The expression of seven proteins increased significantly after cryopreservation. **c** The expression of two proteins decreased significantly after cryopreservation. Differentially expressed (>3-fold) proteins were determined by comparing samples before and after cryopreservation (*P* < 0.05, *n* = 9). The line indicates the landmark of equal levels for before cryopreservation

Spermatozoa samples were analyzed by 2-DE before and after cryopreservation. A total of 285 proteins were detected, and nine proteins were found to be differentially expressed (>3 fold, Table 1 and Fig. 4). The expression of major fibrous sheath protein (AKAP), F1-ATPase complexed with aurovertin B (F1-ATPase), triosephosphate isomerase (TPI), nucleoside diphosphate kinase 7 (NDPK7), NADH dehydrogenase flavoprotein 2 (NDUFV2), F-actin-capping protein subunit beta (CAPZB), and superoxide dismutase 2 (SOD2) were higher after cryopreservation. However, expression of outer dense fiber protein 2 (ODF2) and uncharacterized protein LOC616410 were higher in before cryopreservation (Table 1 and Fig. 4).

**Table 1:** Differentially expressed (>3-fold) proteins of spermatozoa at different steps of cryopreservation

Spot no.	Protein	NCBI no.	MASCOT score^a^	Expression ratio^b^	Search
228	Major fibrous sheath protein chain D, bovine mitochondrial	gi|4588120	88.0	3.87	NCBI
1304	F1-ATPase complexed with aurovertin B	gi|1827812	183.0	12.57	NCBI
2311	PREDICTED: Uncharacterized protein LOC616410	gi|119914715	299.0	0.25	NCBI
4208	Triosephosphate isomerase	gi|61888856	174.0	6.48	NCBI
4418	Nucleoside diphosphate kinase 7	gi|62751773	64.0	6.13	SwissProt
4607	Outer dense fiber protein 2	gi|84000345	290.0	0.46	NCBI
5204	NADH dehydrogenase flavoprotein 2	gi|72004	67.0	5.30	SwissProt
5305	F-actin-capping protein subunit beta	gi|28603770	53.0	5.73	NCBI
7206	Superoxide dismutase, mitochondrial	gi|88853816	74.0	3.92	NCBI

^a^MASCOT scores are −10×log(P), where P is the probability that the observed match is a random event. Individual scores > 40 indicate identity or extensive homology (*P* < 0.05)

^b^Expression ratio is the ratio of relative volume of protein spots with after cryopreservation value to over before

### Western blot

**Fig. 5:**
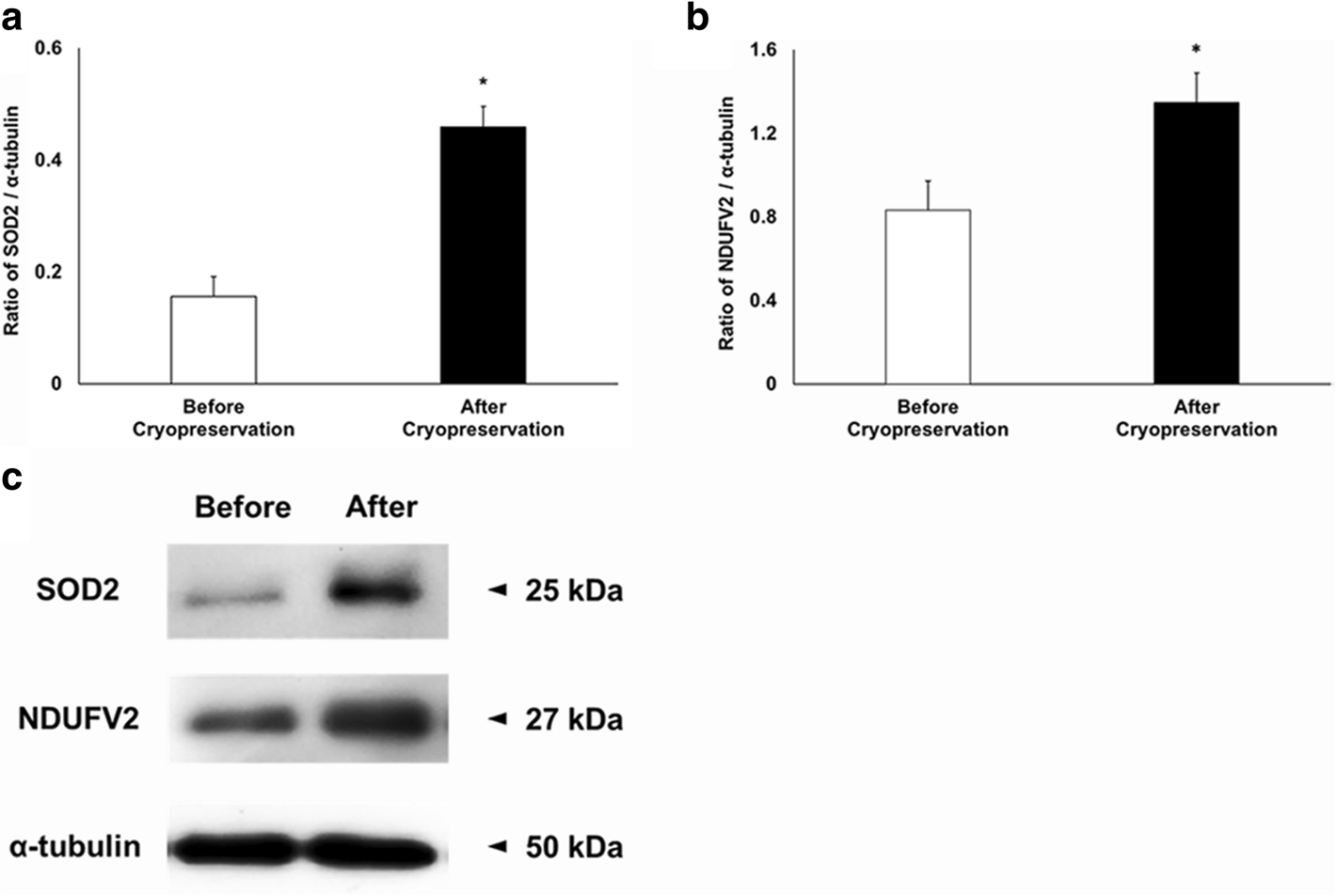
Expression of SOD and NDUFV2 before and after cryopreservation. **a** Ratio of SOD2 to α-tubulin expression before and after cryopreservation. **b** Ratio of NDUFV2 to α-tubulin expression before and after cryopreservation. **c** Expression of SOD, NDUFV2 and before and α-tubulin after cryopreservation. Data are presented as mean ± SEM. (*P < 0.05, *n* = 9)

Western blotting analysis was performed to confirm the 2-DE results. Two differentially expressed proteins were examined using commercial antibodies. SOD2 and NDUFV2 were detected at 25 and 27 kDa, respectively. The densities of SOD2 and NDUFV2 expression were higher after cryopreservation than before (Fig. 5, *P* < 0.05), consistent with the 2-DE results.

### Informatics

**Fig. 6:**
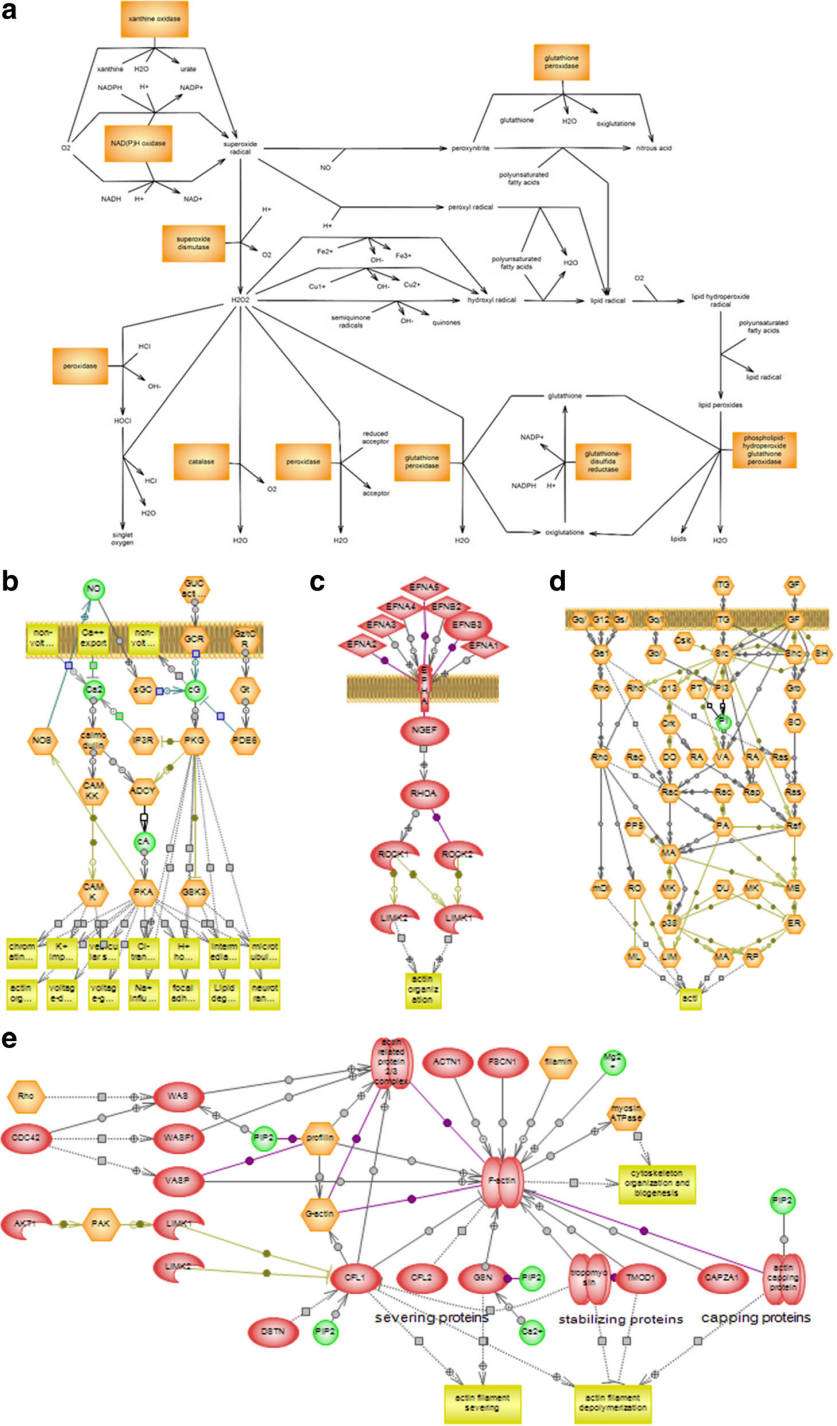
Signaling pathways associated with differentially expressed proteins as identified by Pathway Studio. **a** ROS metabolism is associated with SOD2. **b** Guanylate cyclase pathway is associated with CAPZB, NDPK, and ODF2. **c** EphrinR-actin signaling pathway is associated with CAPZB and ODF2. **d** Actin cytoskeleton regulation associated with CAPZB and ODF2. **e** Actin cytoskeleton assembly is associated with CAPZB

Nine proteins were differentially expressed before and after cryopreservation. They were analyzed using Pathway Studio 9. Five signaling pathways were significantly correlated with four proteins (Fig. 6 and Table 2, *P* < 0.05). CAPZB and ODF2 were correlated with the ephrinR-actin signaling pathway and CAPZB, TPI, and ODF2 were correlated with the Notch pathway (Table 2, *P* < 0.05). The actin cytoskeleton assembly pathway was correlated with CAPZB and actin cytoskeleton regulation was correlated with CAPZB and ODF2 (Table 2, *P* < 0.05). Moreover, the guanylate cyclase pathway was correlated with CAPZB, NDPK, and ODF2 (Table 2, *P* < 0.05).

**Table 2:** Signaling pathways associated with differentially expressed proteins as identified by Pathway Studio

Signaling pathways	Overlapping entities	*P*-value
EphrinR - > Actin Signaling	CAPZB, ODF2	0.005
ROS metabolism	SOD2	0.041
Actin Cytoskeleton Assembly	CAPZB	0.016
Actin Cytoskeleton Regulation	CAPZB, ODF2	0.020
Guanylate Cyclase Pathway	CAPZB, NDPK, ODF2	0.034

## Discussion

Numerous studies have prviously reported that ejaculated and epididymal spermatozoa have differences in sperm maturity, fertility, and sensitivity to freezing. However, in certain situations, it is not possible to collect semen using normal methods, and the cryopreservation of epididymal spermatozoa can be an acceptable alternative. Epididymal spermatozoa can be stored for more than 24 h without loss of fertility [20]. Moreover, a recent study reported that epididymal spermatozoa had a similar fertilizing-capacity as fresh ejaculated spermatozoa using an *in vitro* fertilization system [21]. Therefore, the cryopreservation of epididymal sperm can be a useful assisted reproductive technology for the livestock industry and preserving genetic resources. However, the effects of cryo-stress at the proteome level, especially in epididymal spermatozoa, have not yet fully understood.

During cryopreservation, spermatozoa are exposed to various types of stress such as cold shock, osmotic stress, and ice crystal formation [1, 10]. These environmental changes cause physical and chemical damage to spermatozoa, resulting in changes of various parameters, including viability, motility, motion kinematics, and capacitation status [22].

Motility is one of the most important sperm fertility parameters, and numerous studies have described its essential role in sperm transport to the site of fertilization in the female reproductive tract [12, 23]. Moreover, motion kinematics assessed by CASA provide an accurate representation of sperm movement and its relationship with sperm viability [24]. Our result showed that viability was significantly reduced during cryopreservation. Motility (%), velocity parameters (VCL, VSL, and VAP), and ALH were also significantly reduced. These parameters are closely related to sperm semen quality and fertility [25].

Capacitation refers to the structural and metabolic alterations that allow spermatozoa to fertilize an oocyte [17, 26]. Without capacitation, spermatozoa lack fertilization ability, although they are motile and morphologically normal [27]. Cryopreservation also results in nonfunctional capacitation, i.e., premature capacitated spermatozoa. Premature capacitation causes an aberrant acrosome reaction in spermatozoa [5, 28]. In our study, the AR pattern significantly increased and the F pattern significantly decreased after cryopreservation.

It has been reported that membrane cytoskeletal components are sensitive to temperature and cryoprotectants, which cause damage to sperm surface proteins [5, 10]. Moreover, cryoprotectant toxicity can induce alterations in sperm membrane components [29]. For these reasons, the mechanisms of cryopreservation and the physiological alterations of spermatozoa should be examined at the protein level using sperm proteomic studies. 2-DE is a typical proteomic technique for purifying individual proteins from various samples [12]. Therefore, we studied the mechanisms of cryostress using the 2-DE technique and confirmed the results using western blotting.

It is generally accepted that transcription, translation, and protein synthesis do not occur in mature spermatozoa [30, 31]. However, several studies report that mature spermatozoa have the ability to synthesize proteins [17, 32, 33]. These studies suggest that another possible mechanism, such as post translational modifications is involved in cryostress [33]. We found nine proteins that were differentially expressed before and after cryopreservation (Table 1). Among these nine, two proteins (ODF2 and LOC616410) decreased and seven proteins (SOD2, CAPZB, NDUFV2, NDPK, TPI, F1-ATPase, and AKAP) increased after cryopreservation.

ODF2 is a cytoskeletal structure protein localized in sperm flagella [34]. It functions in the maintenance of sperm structures and movement [35]. ODF could be consider as a marker of male infertility factor and defects of ODF can lead to abnormal morphology and infertility [36]. The absence of ODF also could result in nonfunctional tails and affect sperm movement [33]. ODF2 expression decreased after cryopreservation, and as a result, motility and viability were also found to decrease.

SOD2, a member of the superoxide dismutase family, is an isozyme of superoxide dismutase and an antioxidant [37]. SOD2 can improve cell survival by reducing the level of ROS [38, 39]. In the testis, it reacts with ROS directly and reduces toxicity [40]. During cryopreservation, oxidative stress damages spermatozoa [41]. In our study, SOD2 was extremely highly expressed after cryopreservation. Therefore, it is tempting to speculate that this increased expression reflects a defensive response to protect the spermatozoa against oxidative stress during cryopreservation [42].

CAPZB is a member of the F-actin capping protein family and a major cytoskeletal protein [43]. CAPZB is involved in F-actin function. F-actin depolymerization inhibits capacitation and acrosome reactions [44]. Capping proteins assemble and disassemble filaments in the outer acrosomal membrane during capacitation, and these mechanisms are important for the induction of the acrosome reaction [45]. The higher expression of CAPZB after cryopreservation may be related to capacitation-like changes during cryopreservation. Therefore, the capacitation status results from our study suggest that the increase in CAPZB is related to changes in the AR and F patterns.

NDPK localizes in mitochondria and catalyzes reversible reactions involving a nucleoside triphosphate at the expense of ATP [46]. NDPK is also widely known to regulate transcription and cell proliferation as well as energy metabolism [47]. Sperm motility is closely associated with energy metabolism and excessive metabolic activity could reduce the life span of spermatozoa [18]. We believe that it might be associated with the decreased viability and motility in our study (Fig. 1). Moreover, NDPK has protective effects against oxidative stress [48]. Cryostresses such as oxidative stress and cold shock cause metabolic and functional changes in spermatozoa [49]. In our study, NDPK7 increased after cryopreservation (Fig. 4). This suggests that oxidative stress and changes in energy metabolism occur to protect spermatozoa during cryopreservation.

TPI is an enzyme that promotes the conversion of dihydroxyacetone phosphate to d-glyceraldehyde 3-phosphate [50]. This enzyme plays an important role in sperm metabolism, such as capacitation and the acrosome reaction. Bone et al. [51] have shown that the inhibition of TPI prevents capacitation in rat spermatozoa. The decreased F pattern and increased AR pattern in our capacitation status results might be associated with the high expression of TPI. TPI also induces early capacitation after cryopreservation, resulting in cell death. This protein is highly expressed in spermatozoa that have low motility parameters or poor freezability [52]. In the current study, TPI was highly expressed after cryopreservation (Fig. 4), which may indicate that spermatozoa are damaged during cryopreservation. In addition, our results show that viability and motility decreased (Fig. 1). Moreover, in human sperm, the levels of TPI were higher in asthenozoospermic samples than in normospermic samples [53].

Four of the nine proteins that were differentially expressed before and after cryopreservation were significantly correlated with five signaling pathways (Table 2). These signaling pathways are also associated with sperm functions. The ROS metabolism pathway is associated with SOD2 (Table 2). ROS is necessary for sperm metabolism and various sperm functions such as viability, capacitation and fertility, however high levels of ROS induce oxidative damages to spermatozoa [54]. Oxidative damage causes lower motility, DNA damages, and lipid peroxidation [55]. Membrane phospholipids are related to cell freezability, which affects sperm motility and mitochondrial potential [16, 56]. SOD2 is a major antioxidant, and it reacts with ROS directly to protect against oxidative stress [40]. ROS metabolism is also related the the guanylate cyclase pathway and energy metabolism [57, 58].

The guanylate cyclase pathway plays an important role in Ca^2+^ influx and ROS metabolism [58]. The uptake of Ca^2+^ is a key factor in sperm capacitation and accelerates the acrosome reaction [59]. In the current study, the alterations in capacitation status after cryopreservation may be closely related to this pathway (Fig. 3). Moreover, guanylate cyclase is associated with sperm motility and energy metabolism [60]. Our results show that CAPZB, NDPK, and ODF2 are significantly associated with the guanylate cyclase pathway (Table 2). NDPK has protective effects against oxidative stress and a strong relationship with energy metabolism [47]. The increase in NDPK in our study may have been induced by ROS or excessive energy activity resulting in reduced motility and viability (Fig. 1). Moreover, the increase in SOD2 also can be explained by excessive ROS caused by cryopreservation (Fig. 1, Table 1).

CAPZB was also associated with actin cytoskeleton assembly and actin regulation pathways (Table 2). These pathways are involved in sperm capacitation and the acrosome reaction [61]. Phosphatidylinositol (4, 5)-bisphosphate and phosphatidylinositol (3, 4, 5)-trisphosphate in these pathways play important roles in the regulation of actin polymerization [62]. Sperm capacitation and the acrosome reaction are closely associated with actin polymerization [63]. F-actin in the actin-related pathways plays a major role in the remodeling of actin structure, the acrosome reaction, and male fertility [64]. It is possible that the significant alterations of capacitation status in our results are related to these pathways (Fig. 3).

## Conclusions

In the present study, we compared sperm parameters and the proteome of bovine epididymal spermatozoa before and after cryopreservation. We identified proteins related to cryostress and their associated signaling pathways. To the best of our knowledge, this is the first study to evaluate the effects of epididymal sperm cryopreservation at the proteome level. Our results indicate that proteins could be useful biomarkers for cryostress, and these biomarkers could be used in future studies to identify the mechanism of cryostress responses.

## Abbreviations

2-DE: Two-dimensional electrophoresis
AKAP: Major fibrous sheath protein
ALH: Amplitude of lateral head displacement
AR: Live acrosome-reacted
B: Live capacitated
CAPZB: F-actin-capping protein subunit beta
CASA: Computer-assisted sperm analysis
CTC: Chlortetracycline fluorescence
D: Dead
ESI: Nano-electrospray ionization
F: Live non-capacitated
F1-ATPase: F1-ATPase complexed with aurovertin B
H33258: Hoechst 33258
LIN: Linearity
MS/MS: Tandem mass spectrometry
NDPK7: Nucleoside diphosphate kinase 7
NDUFV2: NADH dehydrogenase flavoprotein 2
ODF2: Outer dense fiber protein 2
PBS: Phosphate-buffered saline
ROS: Reactive oxygen species
RT: Room temperature
SOD2: Superoxide dismutase 2
STR: Straightness
TOF: Time-of-flight
TPI: Triosephosphate isomerase
TYB: Tris–egg yolk buffer
VAP: Verage path velocity
VCL: Curvilinear velocity
VSL: Straight-line velocity
WOB: Wobble
